# Herbal medicine in chronic wounds in Calabria region of Italy: an ethnographic study

**DOI:** 10.3389/fsoc.2025.1638784

**Published:** 2025-10-24

**Authors:** Davide Costa, Raffaele Serra

**Affiliations:** ^1^Department of Medical and Surgical Sciences, Magna Graecia University of Catanzaro, Catanzaro, Italy; ^2^Interuniversity Center of Phlebolymphology (CIFL), “Magna Graecia” University, Catanzaro, Italy

**Keywords:** herbal medicine, chronic wounds, Calabria, Italy, ethnographic study, ethnomedicine

## Abstract

**Introduction:**

Traditional herbal medicine remains a vital, though often overlooked, component of chronic wound management in rural and underserved areas of southern Italy. In Calabria, this enduring practice reflects both cultural continuity and systemic healthcare gaps.

**Methods:**

An ethnographic study was conducted involving 120 patients attending a vascular surgery clinic in Catanzaro, Calabria. Data were collected through semi-structured interviews, participant observation, and field notes, focusing on the use and transmission of herbal remedies for chronic ulcers.

**Results:**

The findings reveal that older women, particularly grandmothers and mothers, are the primary custodians of local ethnomedical knowledge. Remedies such as *Achillea millefolium* poultices and *Citrus bergamia* decoctions are employed to treat various types of chronic wounds, including venous, arterial, and diabetic ulcers. These treatments are adapted to a vernacular taxonomy of wound severity and are often accompanied by symbolic or ritual meanings. Despite biomedical availability, patients—especially those from low-income or rural settings—continue to use plant-based therapies due to limited healthcare access, long waiting lists, and a perceived over-medicalization of care. Gender dynamics were evident, with women maintaining knowledge transmission within the household and men engaging more in self-treatment related to occupational injuries.

**Discussion:**

In this Calabrian context, herbal medicine functions not as an alternative, but as a parallel and coexisting system of care. It offers cultural affirmation, therapeutic autonomy, and practical solutions amid healthcare challenges. Documenting such practices deepens our understanding of medical pluralism and highlights the need to integrate local voices into ethnobotanical research.

## 1 Introduction

Chronic wounds, defined as wounds that fail to progress through the normal stages of healing in an orderly and timely manner, present a significant challenge to healthcare systems worldwide. These wounds, often persisting beyond 4–12 weeks despite appropriate treatment, are associated with considerable morbidity and healthcare costs (Bowers and Franco, 2020; Serra et al., 2015). These wounds, often caused by conditions such as chronic venous insufficiency (CVI), lymphatic insufficiency, chronic limb ischemia (CLI), diabetes, skin tears, or prolonged pressure, can lead to severe complications, including infections and amputations, thereby reducing patients’ quality of life. Specifically, CVI may manifest as significant skin changes, including venous eczema and lipodermatosclerosis, as well as active chronic venous ulcers (CVUs), which represent a serious form of skin tissue damage. CLI can result in localized or extensive arterial ischemic ulcers (IAUs), and in some patients, both arterial and venous wounds may even occur simultaneously in the same patient, referred to as mixed arterial and venous ulcers (MAVLUs). Furthermore, diabetic patients may experience diabetic foot ulcers (DFUs), which contribute to increased overall morbidity in this population (Serra et al., 2017; De Franciscis et al., 2014; Serra et al., 2018; Costa et al., 2022; Serra et al., 2014; Serra et al., 2015; Ammendola et al., 2017).

The increasing prevalence of chronic wounds has prompted a search for effective and accessible treatments, leading to a renewed interest in herbal medicine practices (Monika et al., 2022; Maver et al., 2015; Shedoeva et al., 2019).

Among traditional medicine, encompassing the knowledge, skills, and practices based on the theories, beliefs, and experiences indigenous to different cultures, has been utilized for centuries to treat various ailments, including wounds (Elendu, 2024; Reyes-García, 2010; Hill, 2009).

Herbal remedies, in particular, have played a pivotal role in wound management due to their antimicrobial, anti-inflammatory, and healing properties (Polerà et al., 2019; Gasbarro et al., 2013).

Thus, medical humanities are useful to examine the social dimensions of herbal medicine use. The knowledge of medicinal plants is often passed down through generations, and herbal remedies for chronic wounds are embedded in local cultural practices, shaping social relations and community identity (Xu et al., 2023). Moreover, the increasing integration of traditional and modern healthcare systems in many countries has led to the establishment of ethnobotanical research, which bridges scientific knowledge with traditional healing practices (Duke and Ayensu, 1985). The herbal medicine plays a multifaceted role in the treatment of chronic wounds, with its significance shaped by both pharmacological properties and cultural contexts. By employing sociological and anthropological perspectives, it is possible to understand the complex relationships between people, plants, and healing practices, providing insights into how traditional and modern medical systems can collaborate for improved wound care management (Xu et al., 2023).

The management of chronic wounds presents an ongoing challenge across both biomedical and traditional medical systems. Biomedical approaches to wound care are typically guided by standardized protocols involving antiseptics, debridement, antibiotics, and advanced therapies such as compression therapy, skin grafts, or bioengineered dressings (Frykberg and Banks, 2015). These methods are supported by clinical evidence and are widely used in hospital settings; however, they often require specialized care, are resource-intensive, and may be difficult to access in rural or economically underserved areas (Gray et al., 2018).

In contrast, traditional medical systems—particularly those rooted in ethnobotanical knowledge—approach wound healing through culturally embedded practices that incorporate locally available plants with known or perceived antimicrobial, anti-inflammatory, and regenerative properties (Shedoeva et al., 2019; Maver et al., 2015; Monika et al., 2022). Rather than isolating biological mechanisms, these practices often involve holistic understandings of illness, treating the wound not just as a physical ailment but as a disturbance of bodily, environmental, or spiritual balance. Remedies are typically administered through poultices, decoctions, and infusions, often prepared by caregivers within the family or community (Dorai, 2012).

While biomedical and traditional medicine are sometimes presented as opposing paradigms, increasing research suggests they can be complementary rather than contradictory. Several medicinal plants used in traditional systems have demonstrated pharmacological efficacy in laboratory and clinical studies (Polerà et al., 2019), supporting calls for more integrative approaches. However, there also remain tensions—such as differences in epistemologies, regulation, and professional authority—that complicate integration.

In several cultural contexts, these two systems often coexist in a hybrid form. Patients move fluidly between formal healthcare providers and informal, culturally rooted care practices. This coexistence is not merely pragmatic but shaped by structural constraints (e.g., healthcare access), cultural identity, and trust in ancestral knowledge. In fact, the Global Centre for Traditional Medicine in Jamnagar Gujarat – India – showcases a defined move to integrating the two systems (Singh et al., 2012).

Understanding how these systems interact in everyday healing practices is crucial for designing more inclusive and effective healthcare strategies in regions where biomedical care may be perceived as distant or insufficient (Jama et al., 2024).

For clarity and consistency, key terms and concepts employed throughout this article—particularly those related to traditional and ethnomedical practices—are defined in the Table 1.

**Table 1:** Glossary list.

Term	Definition
Herbal medicine	It is the use of plant-derived substances with therapeutic properties or healing potential to prevent, treat, or manage disease. It includes the use of whole plants, plant parts, or plant extracts prepared through traditional or modern pharmacological methods.
Traditional medicine	It refers to the sum total of knowledge, skills, and practices based on the theories, beliefs, and experiences indigenous to different cultures, used in the maintenance of health, as well as in the prevention, diagnosis, improvement, or treatment of physical and mental illness.
Herbal medicine practices	They encompass the rituals, techniques, and therapeutic uses involving plant-based remedies within specific cultural or healing systems, including collection, preparation, dosage, and administration of medicinal plants.
Medicinal plants	They are plants containing bioactive compounds that are used for therapeutic purposes in traditional or modern medical systems. These may include roots, leaves, seeds, flowers, or extracts with proven or presumed pharmacological effects.
Traditional healing practices	They are culturally embedded therapeutic modalities employed by healers or community members that may involve herbal remedies, spiritual rituals, divination, massage, or symbolic interventions, rooted in the community’s belief systems and cosmology.
Ethnomedical knowledge	It refers to culturally specific systems of health-related understanding, including concepts of disease etiology, diagnosis, treatment, and healing, developed within the context of a particular ethnic or cultural group.
Matrilineal healing practices	They are healing traditions and knowledge systems that are transmitted through the female line, often within families or clans, where women act as custodians of specific medicinal, ritual, or therapeutic techniques.
Ethnomedical landscape	It refers to the symbolic, ecological, and social space in which medical beliefs and practices are embedded, shaped by local geography, plant availability, cosmologies, and health-related traditions.
Vernacular healing practices	They are locally developed, non-institutional medical practices, often transmitted orally and informally, that rely on folk knowledge, indigenous classifications, and accessible remedies without necessarily conforming to formalized traditional or biomedical systems.
Ethnomedical system	It is a structured, culturally coherent set of beliefs and practices concerning health and illness, including etiology, diagnostic procedures, healing rituals, and therapeutic techniques, specific to a social group.
Ethnobotanical knowledge	It is the culturally transmitted understanding of plant uses within a community, especially relating to medicinal, nutritional, symbolic, and ritual applications, based on long-term empirical interaction with the local environment.
Ethnomedicinal knowledge	It is a subset of ethnobotanical knowledge that focuses specifically on the medicinal uses of plants, including plant identification, preparation methods, dosage, indications, and contraindications, as practiced within a cultural or traditional medical system.

## 2 The aim of the study

This ethnographic study aims to bridge the existing knowledge gap by systematically documenting whether patients with chronic ulcers use herbal medicines and other forms of traditional medicine in the management and treatment of chronic ulcers, the possible method of preparation of the medicines and the cultural meanings that patients attribute to this type of treatment.

## 3 Context

In the context of Italy, the region of Calabria offers a rich tapestry of traditional healing practices deeply rooted in its history and culture. Ethnobotanical studies have documented the use of various medicinal plants in this region. These plants have been employed to treat a range of ailments, including wounds, reflecting a profound understanding of the local flora’s medicinal properties. Moreover, recent scientific investigations have begun to validate the efficacy of herbal Calabrian remedies (Passalacqua et al., 2007; Patti et al., 2025). For example, traditional foods, such as honey have been suggested as potential therapeutic applications for chronic wounds (Governa et al., 2019), thus underscoring the importance of integrating traditional knowledge with modern scientific research to develop effective wound care strategies. Despite the rich heritage and potential benefits of herbal medicine in Calabria, there remains a scarcity of comprehensive ethnographic studies exploring their application in chronic wound management. Understanding the traditional practices, cultural beliefs, and local perceptions surrounding wound care in this region is crucial for several reasons. Firstly, it can lead to the identification of novel therapeutic agents derived from local plants. Secondly, it can inform culturally sensitive healthcare interventions that resonate with the local population’s beliefs and practices, thereby enhancing patient compliance and outcomes. Lastly, it contributes to the preservation of intangible cultural heritage, ensuring that valuable traditional knowledge is not lost to future generations (Krippner et al., 2011; Xu et al., 2023).

Southern Italy—particularly the Calabria region—has experienced a slow but persistent transformation in the relationship between healthcare systems, local communities, and traditional healing practices. This period has seen a resurgence in ethnomedical knowledge, particularly concerning chronic wound management, due to the fact the region facing systemic underfunding, rural depopulation, and healthcare disparities (Gallante et al., 2011). Calabria remains one of Italy’s most underserved regions, marked by limited access to specialized medical services such as vascular care, especially in mountainous and peripheral rural areas (Costa et al., 2025). Against this backdrop, herbal medicine has emerged not merely as an alternative therapeutic option but as a coexisting and culturally legitimized system of care (Passalacqua et al., 2007; Patti et al., 2025). Parallel to this informal medical sphere is the formal biomedical system, represented by vascular clinics, general practitioners, and hospital services. In Calabria, this system is often perceived by participants as distant, overburdened, and inaccessible, particularly in rural zones with high elderly populations (Brienza et al., 2002). The perception of medical over-specialization and bureaucratic delays drives many patients to seek more immediate and culturally resonant care through traditional means. In this ethnomedical landscape, one can also identify the presence of intermediary figures: pharmacists, nurses, and even local herbalists who—while situated within or adjacent to the biomedical model—facilitate informal exchanges of plant knowledge and reinforce traditional practices through tacit approval or discreet collaboration. This convex arrangement—where traditional knowledge-holders, patients, informal intermediaries, and the biomedical system all intersect—produces a hybrid model of healthcare (Byrne et al., 2022). The ethnographic data demonstrate that this hybridity is not simply a result of resistance to biomedicine, but rather a negotiated response to structural deficiencies, cultural identity, and therapeutic pragmatism (Maizes et al., 2009). Just as political parties and social movements interact dynamically in the field of climate activism, in Calabria, vernacular healing practices and institutional medicine continually redefine their boundaries through everyday interactions and community narratives. Additionally, socioeconomic and political marginalization plays a key role in shaping the persistence of herbal medicine (Costa-Font and Sato, 2025). Rural Calabrian communities are often excluded from national public health discourse, echoing a broader trend of healthcare inequity across southern Italy. This marginalization fosters a localized form of therapeutic autonomy, where the use of native plants, knowledge sharing within families, and gendered caregiving roles become forms of resistance and resilience in the face of systemic neglect (Taroni, 1861). The Calabrian ethnomedical system operates within a complex social ecology—one where traditional healing, gender, local epistemologies, and biomedical infrastructures continuously overlap and influence each other. This analysis helps reposition herbal medicine not as a relic of the past, but as a living cultural system responding dynamically to contemporary healthcare challenges (Patti et al., 2025; Krippner et al., 2011).

**Figure 1:**
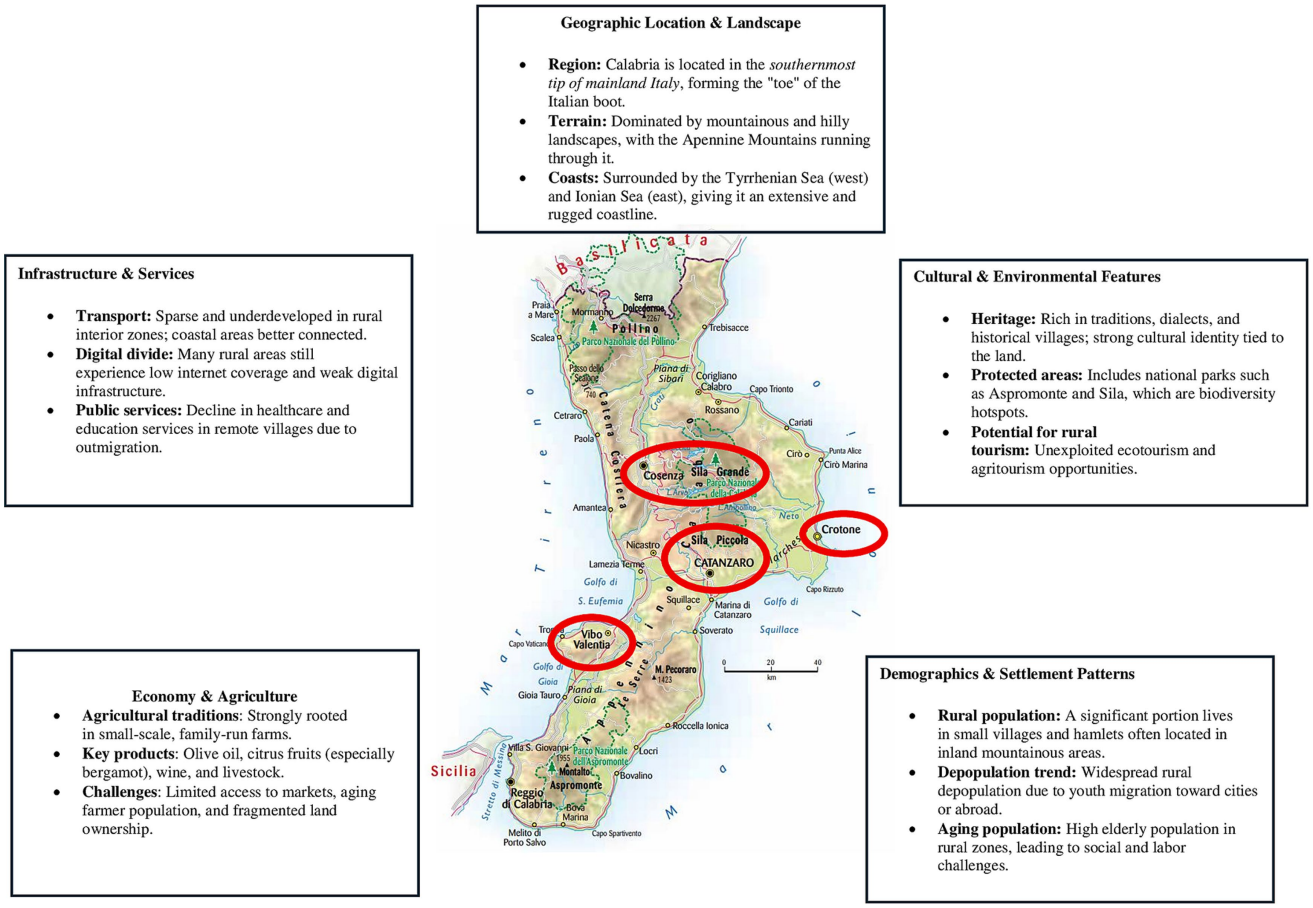
Map of the rural context of Calabria and the city most involved.

To better understand the context of the empirical case study, a map has been created (Figure 1) highlighting all the main characteristics of the Calabria region, with the cities most involved in the research circled in red.

## 4 Methodology

This article is grounded in an ethnographic research approach, which is particularly suited for exploring the cultural, symbolic, and practical dimensions of traditional medicine in specific social settings. Ethnography provides tools for in-depth engagement with communities, enabling the documentation of local knowledge systems and therapeutic practices as they are lived and experienced (Shah, 2017). Participant observation and semi-structured interviews, key methods of ethnographic inquiry, allow researchers to immerse themselves in the field and follow social dynamics, meaning-making processes, and interactions surrounding healthcare choices (Morgan-Trimmer and Wood, 2016).

The present study is based on long-term, multi-sited ethnographic fieldwork conducted in Calabria, Southern Italy, between January 2019 and November 2024. The research focused on patients with chronic wounds attending the vascular surgery clinic of the Dulbecco University Hospital in Catanzaro.

Eligibility required the presence of one or more chronic wound conditions and the use of herbal medicine for wound management, along with the patient’s willingness and ability to participate in ethnographic data collection, which included semi-structured interviews and participant observation. All participants were required to be 18 years of age or older. Patients were excluded if they did not use herbal remedies, declined to participate, were unable to provide informed consent, or had cognitive or communication impairments that could interfere with the qualitative research process.

Regarding the sex of the patients 54 were males (45%) and 66 were females (55%), aged between 61–92 years with a mean age of 71.38 years. The participants were divided in four groups. The first group consisted of 89 individuals (74.17%) with skin changes of venous origin (venous eczema and lipodermatosclerosis) and/or chronic venous leg ulcers (CVLUs), 35 were males (39.33%) and 54 were females (60.67%). The second group consisted of 10 individuals (8.33%) with arterial leg ulcers (ALUs). Seven were males (70%) 3 females (30%). The third group consisted of 12 individuals (10%) with mixed arterial and venous ulcers (MAVUs). Seven were males (58.33%) and 5 were females (41.67%). The fourth group consisted of 9 individuals (7.5%) with diabetic foot ulcers (DFUs). Five were males (55.56%) and 4 were females (44.44%).

The ethnographic engagement included semi-structured interviews, participant observation in clinical and community settings, and the production of detailed field notes, enabling a comprehensive understanding of herbal medicine usage in the management of chronic ulcers. The study particularly sought to explore the cultural logic of wound classification, plant selection, and preparation methods, as well as the gendered transmission of ethnobotanical knowledge.

The first author, an experienced in qualitative and ethnographic research, led the fieldwork, with prior research experience in Southern Italy. His familiarity with local dialects and cultural norms facilitated rapport-building with elderly informants and access to gender-specific domains of knowledge. Although not a practitioner of herbal medicine, the researcher shared an interest in local health traditions and was transparent about the study’s aims. Informed consent was obtained from all participants. Interviews were conducted in Italian, recorded, and professionally transcribed. Participants were assigned pseudonyms to ensure confidentiality. The methodology was reviewed and approved by the Institutional Review Board of the Interuniversity Center of Phlebolymphology (CIFL) (Reference: ER. ALL.2018.73A). The multi-sited nature of the fieldwork allowed for comparative insights across various sub-regions of Calabria, capturing regional and gendered variations in healing practices. Participant observation included household visits where remedies were prepared, informal conversations with patients and caregivers, and observation of the therapeutic use of plants in everyday contexts. While men were often observed preparing poultices for work-related injuries, women—especially grandmothers and mothers—emerged as the primary transmitters and custodians of plant-based healing knowledge.

The interviews were conducted in Italian and were recorded end consequently professionally transcribed, and the duration was among 45 min. The research materials were undertaken by the first author and supplementary analysis was undertaken at the conclusion of the field research. Pseudonyms are used in this paper for all participants to maintain confidentiality.

The medicinal plants identified in our study are summarized in Table 2, in which botanical taxon and family, local name, English common name, used parts, type of preparation and treated wound or skin changes have been provided for each plant.

**Table 2:** Medicinal plants, type of preparation, type of lesions and patients.

Botanical taxon and family	Local name	English common name	Used parts	Preparation	Treated wound-skin changes	Male	Female	Frequency of citation (number)	Frequency of citation (%)
*Achillea millefolium*, Asteraceae	Achillea	Yarrow	Flowers	Pulverized	Chronic venous ulcers	2	2	4	3.33
*Calendula arvensis*, Compositae	Calentula	Marigold	Flower heads	Decoction	Arterial ischemic ulcers; Diabetic foot ulcers	4	0	4	3.33
*Castanea sativa*, Fagaceae	Castagnaru	Chestnut tree	Leaves	Decoction	Venous eczema; Lipodermatosclerosis	0	1	1	0.83
*Cichorium intybus*, Compositae	Cicoria	Chicory	Leaves	External use	Chronic venous ulcers	1	4	5	4.17
*Citrus bergamia*, Rutaceae	Bergamotto	Bergamot orange	Esssential oil	Diluited	Diabetic foot ulcers; Arterial ischemic ulcers; Mixed arterial and venous ulcers	3	3	6	5
*Cucumis sativus*, Cucurbitaceae	Citrulu	Cucumber	Ripe fruit	Squashed pulp	Venous eczema; Lipodermatosclerosis	1	4	5	4.17
*Daucus carota* subspecies sativus, Umbelliferae	Carota	Carrot	Root	Poultice	Venous eczema; Lipodermatosclerosis	2	1	3	2.5
Equisetum arvese, Equisetaceae	Erba cavallina	Common horsetail	Aerial part	Infusion	Chronic venous ulcers	3	1	4	3.33
*Eucalyptus globulus*, Myrtaceae	Calipto	Blue gum	Leaves	Decoction	Diabetic foot ulcers	1	0	1	0.83
*Juglans regia*, Juglandaceae	Nucara	Common walnut	Leaves	Infusion	Chronic venous ulcers	3	1	4	3.33
*Lavandula angustifolia*, Labiatae	Spicanardu	Lavender	Flowery tops	Decoction	Arterial ischemic ulcers; Diabetic foot ulcers	1	1	2	1.67
*Lonicera caprifolium*, Caprifoliaceae	Caprifoglio	Honeysuckle	Leaves	Crushed	Venous eczema; Lipodermatosclerosis; Chronic venous ulcers	2	1	3	2.5
Loranthus europaeus, Loranthaceae	Vischio	Loranthus	Entire plant	Powder	Venous eczema; Lipodermatosclerosis	3	1	4	3.33
*Marrubium vulgare*, Labiatae	Menta sarbaggia	White horehound	Leaves	Infusion	Venous eczema; Lipodermatosclerosis	1	4	5	4.17
Melilotus italica, Leguminosae	Lupinella	Italian melilot	Seeds	Infusion	Venous eczema; Lipodermatosclerosis; Chronic venous ulcers	3	1	4	3.33
*Morus nigra*, Moraceae	Cizu	Black mulberry	Bark or Lymph	Decoction or Local application	Venous eczema; Lipodermatosclerosis	1	6	7	5.83
Ocymum basilicum, Labiatae	Vasalicò	Basil	Leaves	Fresh	Venous eczema; Lipodermatosclerosis	1	3	4	3.33
*Olea europaea*, Oleaceae	Olivaru	Olive tree	Leaves	Decoction	Arterial ischemic ulcers; Chronic venous ulcers; Mixed arterial and venous ulcers; Diabetic foot ulcers	3	1	4	3.33
*Opuntia ficus-indica*, Cactaceae	Pitta e ficu ndiana	Prickly pear cactus	Pad or Juice	Roasted or Extracted juice	Chronic venous ulcers	0	4	4	3.33
*Parietaria officinalis*, Urticaceae	Erba e’ vientu	Pellitory-of- the- wall	Aerial part	Decoction	Arterial ischemic ulcers; Chronic venous ulcers; Mixed arterial and venous ulcers; Venous eczema; Lipodermatosclerosis	2	1	3	2.5
*Passiflora incarnata*, Passifloraceae	Fiore della passione	Passion flowers	Aerial part	Compresses with infusion	Chronic venous ulcers	1	1	2	1.67
Petroselium crispum, Umbelliferae	Petrusinu	Parlsey	Leaves	Poultice	Chronic venous ulcers; Diabetic foot ulcers	1	6	7	5.83
Phaselous vulgaris, Leguminosae	Fagioli	Been	Seeds	Squashed	Venous eczema; Lipodermatosclerosis	1	3	4	3.33
*Plantago major*, Plantaginoceae	Frunna	White man’s footprint	Leaves, Aerial part	Crushed	Arterial ischemic ulcers; Chronic venous ulcers; Diabetic foot ulcers	2	4	6	5
*Prunus dulcis*, Rosaceae	Amendula	Almond	Fruit pulp	Squashed pulp	Venous eczema; Lipodermatosclerosis	1	1	2	1.67
*Ricinus communis*	Erva ‘e latti	Castor bean	Seeds	Cold pressig	Venous eczema; Lipodermatosclerosis	1	1	2	1.68
*Rosa canina*, Rosaceae	Rosa sarbaggia	Rosehip	Bud and Leaves	Infusion	Chronic venous ulcers	1	1	2	1.69
*Rubus ulmifolius*, Rosaceae	Ruvettu	Elmleaf blackberry	Leaves	Decoction	Diabetic foot ulcers	3	0	3	2.5
*Salvia officinalis*, Labiatae	Sarbia	Sage	Leaves, Flowers	Decoction	Arterial ischemic ulcers; Chronic venous ulcers; Mixed arterial and venous ulcers; Diabetic foot ulcers	2	3	5	4.17
*Scrophularia canina*, Scrophulariaceae	Erva lupara	Dog figwort	Aerial part	Boiled	Arterial ischemic ulcers; Chronic venous ulcers;	1	3	4	3.33
Viola, Violaceae	Viola	Viola	Leaves	Crushed	Chronic venous ulcers	1	1	2	1.67
*Vitis vinifera*, Vitaceae	Vite	Common grape vine	Leaves	Decoction	Arterial ischemic ulcers; Chronic venous ulcers	2	2	4	3.33

Fieldnotes were systematically analyzed following the approach of Emerson et al. (1995), involving two key phases: (i) an initial open coding stage, where recurring themes such as gender roles, healthcare access, and symbolic plant use were identified; and (ii) a second stage of memo-writing, enabling the interpretation of underlying meanings and tensions in the data. This dual-stage coding process facilitated the recognition of both shared patterns and local divergences in ethnomedical practices. The methodology also drew from Falzon’s (2009) concept of multi-sited ethnography, enabling the tracing of herbal knowledge across different sociomedical contexts—from clinics to kitchens, from family storytelling to lived patient care. Through this lens, the study investigated not only what plants were used, but how, why, and by whom they were prepared and applied—paying close attention to narratives of empowerment, resourcefulness, and cultural continuity in the face of systemic healthcare limitations. The ethnographic methodology employed in this study offers a rich, context-sensitive account of how herbal medicine persists and evolves in Calabria. It foregrounds the voices of patients and caregivers, particularly women, and sheds light on how cultural knowledge systems interact with healthcare structures, socioeconomic conditions, and therapeutic efficacy.

## 5 Findings and discussion

**Figure 2:**
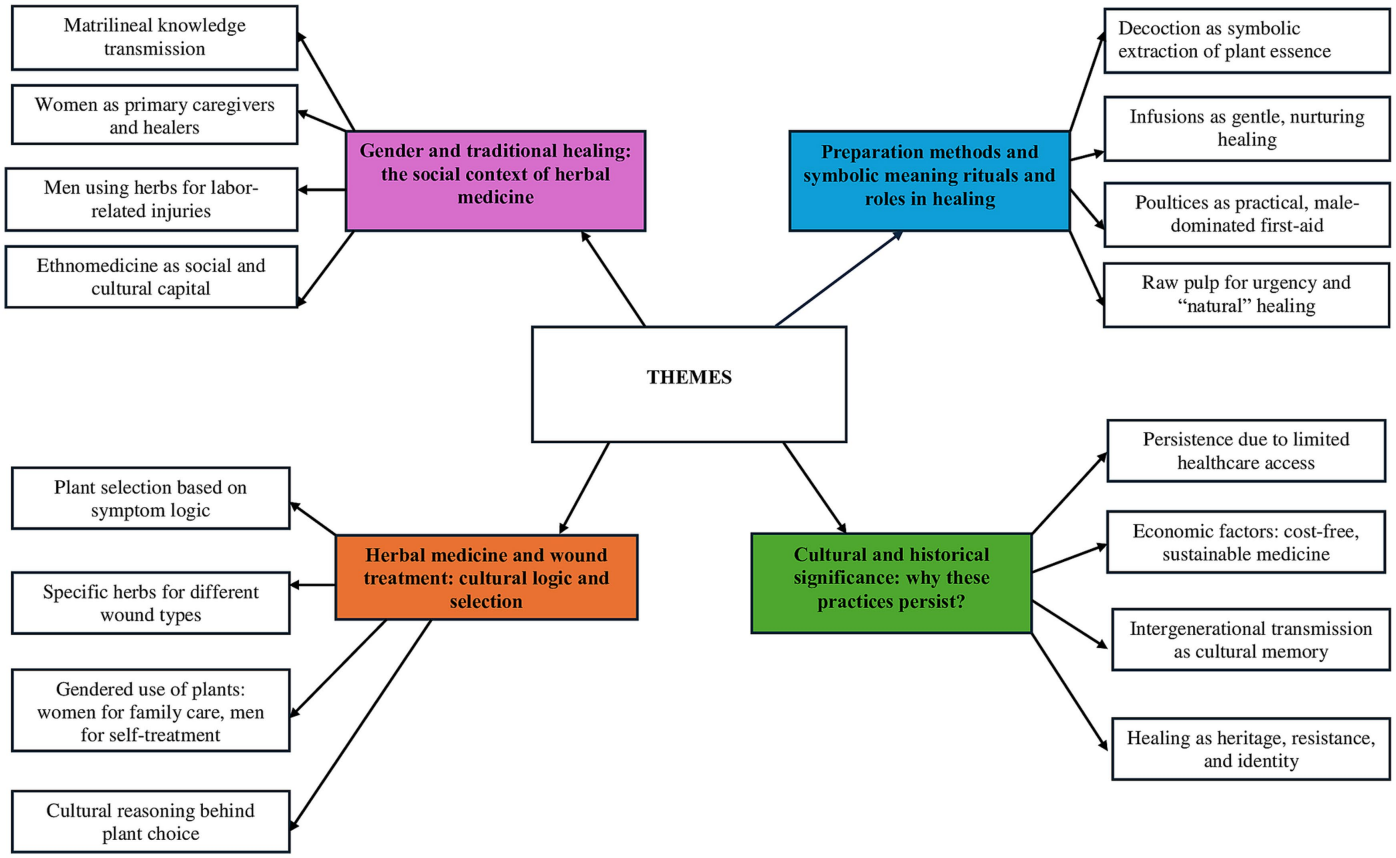
Thematic diagram.

The data analysis revealed four main themes and different subthemes. Figure 2 illustrates the thematic hierarchy, detailing each theme along with its corresponding subthemes.

### 5.1 Gender and traditional healing: the social context of herbal medicine

In Calabria, as in many Mediterranean rural societies, women are the primary custodians of ethnomedicinal knowledge (La Rosa et al., 2021). The dataset reflects a higher frequency of female engagement (132 citations) compared to male engagement (54 citations), suggesting that women play a dominant role in administering and transmitting traditional healing practices.

While men were often observed preparing poultices for work-related injuries, women—especially grandmothers and mothers—emerged as the primary transmitters and custodians of plant-based healing knowledge.

This gender disparity aligns with broader anthropological findings because women, particularly elderly caregivers, are often responsible for treating chronic conditions within families, including venous ulcers, diabetic foot ulcers, and ischemic wounds (Swartzell et al., 2023).

“*To tell the truth, it was my mother-in-law who explained the use of certain herbs for my ulcers, telling me how her grandmother passed this knowledge down to her mother, and then to her… She was very proud to hold this almost secret and magical knowledge*.” (Maria, female, 74 years old)

The knowledge of herbal medicine is typically matrilineal, passed down through generations from mothers and grandmothers. For example, in Hmong communities in Laos, female herbalists pass down their extensive understanding of medicinal plants to younger women, highlighting the gendered nature of this knowledge system. This practice underscores the importance of women in maintaining and disseminating ethnomedical traditions (Dubost et al., 2019).

Traditional medicine use may persist due to limited access to healthcare, financial constraints, or a deep-rooted belief in natural remedies as a holistic alternative to pharmaceuticals. In fact, turn to herbal medicine due to various barriers that make conventional healthcare difficult to access (Gallante et al., 2011; Passalacqua et al., 2007; Patti et al., 2025; Costa et al., 2025). One of the most significant obstacles is the high cost of medical care. For those in low-income communities, the expenses associated with hospital visits, prescription medications, and insurance can be overwhelming. Research by James et al. (2018) highlights that high out-of-pocket costs often push people toward more affordable herbal alternatives. Moreover, the key informants explained how in rural areas, the lack of vascular clinic and trained professionals pushes individuals toward traditional remedies. Cultural beliefs further reinforce this preference, as traditional healing practices are deeply rooted in many societies (Young, 1983; Hill, 2009). Additionally, concerns over side effects and over-medicalization lead some to perceive herbal medicine as a safer alternative (Kaczmarek, 2019; Frawley, 2025). Together, these factors drive the continued reliance on traditional remedies worldwide.

Specifically, chronic lower extremity ulcers generally do not progress through the healing process in a timely manner and have become a major challenge to healthcare systems worldwide. These ulcers, despite appropriate treatment, last on average 12–13 months, recur in up to 60–70% of patients, and can lead to loss of function and decreased quality of life (Frykberg and Banks, 2015). Therefore, the problem of patient dissatisfaction with current treatments, which do not adequately address the problems associated with chronic wounds, pushes patients to resort to alternative treatments, such as herbal medicine. Herbal treatment also gives the patient greater management autonomy compared to conventional treatments, which are perceived as exclusively expert-led. Furthermore, herbal medicine use may persist also due to limited access to healthcare, financial constraints, or a deep-rooted belief in natural remedies as a holistic alternative to pharmaceuticals (Costa et al., 2025).

*“Treatments for venous ulcers are incredibly expensive, which is why I turned to medicinal herbs! Plus, there aren’t many healthcare facilities where I live, and the waiting lists for an appointment are long. So, what am I supposed to do? I don’t have a job… Herbal remedies have given me an alternative—sustainable and completely free!”* (Antonio, male, 61 years old)

Interestingly, while women dominate the ethnomedical landscape, men’s citations (though fewer) indicate engagement in herbal practices, especially for self-care or labor-related injuries. At this regard research suggests that men, particularly those in physically demanding jobs such as farming or construction, turn to herbal medicine for pain relief, wound healing, and stamina enhancement (Pacheco-Cobos et al., 2010). In many cultures, men seek out specific herbal treatments for musculoskeletal injuries, often relying on knowledge acquired through personal experience or male elders rather than formal ethnomedical training (Van Andel et al., 2015).

*“At first, I saw using medicinal herbs as something for women, but over time, I learned to use them—especially when I get injured while working in the fields. With my diabetes, the risk of complications is high, so I rely on them often.”* (Francesco, male 79 years old).

Thus, the presence of men in the dataset may signal an evolving dynamic where herbal medicine is no longer solely a female domain but part of a broader cultural heritage. At this regards we remember that in many cultures, women are the primary custodians of herbal knowledge, serving as healers and midwives. For instance, among the Tuareg people, women herbalists play a crucial role in health practices, utilizing their knowledge of local flora to treat various ailments. Their expertise is not only a means of healthcare but also a way to assert social status and influence within their communities (Rasmussen, 1998).

The ethnographic fieldwork revealed the presence of multiple intersecting actors shaping the use and transmission of herbal knowledge (Niemeyer et al., 2013). Central among them are elderly women, particularly mothers and grandmothers, who function as custodians of matrilineal healing practices. Their position in the household allows them to bridge intimate caregiving roles with broader community-based knowledge transmission. This gendered pattern of authority reflects a structure where healing is not only a practical response to chronic wounds but also a form of cultural stewardship. Alongside these women stand the patients themselves, many of whom engage in self-treatment or peer-assisted healing, especially in cases where biomedical options are economically or geographically inaccessible (Odubo et al., 2024). Elderly men, particularly former agricultural workers, appear more frequently in the data as users of specific herbal treatments for labor-related injuries and diabetic complications. However, the knowledge of how to prepare and apply these remedies often still derives from women in the household or community (Wilentz, 2000).

### 5.2 Herbal medicine and wound treatment: cultural logic in selection

Wounds and skin-related conditions in Calabria’s traditional medicine are primarily treated with plants that possess antimicrobial, anti-inflammatory, and circulatory-enhancing properties. The dataset reveals patterns in how these plants are chosen.

*“You use yarrow when the skin gets red and itchy—it helps draw out the bad blood. That’s what my mother always said. It clears the swelling and brings out the ‘calore’ [heat] from under the skin. We used to boil it and then make compresses—warm, never too hot. You leave it on until the color starts to change, and then you know it’s working. I’ve done this since I was a girl. Back then, we didn’t go to the doctor unless it was serious. These herbs were what we had—and they still work today.”* (Concetta, Female, 72 years old)

In this study, for skin changes of venous origin (venous eczema and lipodermatosclerosis) and Chronic Venous Leg Ulcers (CVLUs), the most cited treatments were *Achillea millefolium* (Yarrow), *Cichorium intybus* (Chicory), *Cucumis sativus* (Cucumber), *Equisetum arvense* (Common horsetail), *Juglans regia* (Common walnut), *Loranthus europaeus* (Loranthus), *Marrubium vulgare* (White horehound), *Melilotus italica* (Italian melilot), *Morus nigra* (Black mulberry), *Ocimum basilicum* (Basil), *Olea europaea* (Olive tree), *Opuntia ficus-indica* (Prickly pear cactus), *Petroselinum crispum* (Parsley), *Phaseolus vulgaris* (Bean), *Plantago major* (White man’s footprint), *Salvia officinalis* (Sage), *Scrophularia canina* (Dog figwort), and *Vitis vinifera* (Common grapevine).

In the context of cultural reasoning, these clinical conditions arise from impaired venous return, a common issue among elderly women and individuals with sedentary habits or jobs (Costa, 2023). Most of these herbs are valued for their antibacterial and tissue-regenerating properties, which make them effective in wound healing and infection control.

*When my legs swell, and the skin turns dark, I use mulberry leaves—my grandma taught me that. She would pick them fresh in the early morning, when the dew was still on them, and then boil them gently. The water turns a bit green, and we’d soak cloths in it to wrap around the legs. It brings relief to the heaviness and helps the skin breathe again. She said the mulberry has ‘sangue buono’ [good blood] inside, and that it cleans the veins. Even now, I follow the same steps. I don’t trust creams from the pharmacy—too many chemicals. This is natural, and it’s part of our way.”* (Anna, female, 82 years old)

Moreover, skin conditions often involve discoloration, inflammation, and chronic itching. The aforementioned plants also provide anti-inflammatory and soothing effects.

Considering gender association, higher female engagement suggests that women treat both themselves and male family members, reinforcing their role as household healers.

*“I treat my husband’s sores the same way I treat mine—it’s the same plants, just more compresses for him because his legs are worse,”* (Giovanna, female, 76 years old)

The high prevalence of venous abnormalities among women suggests that women may have discovered treatments through personal experience before applying them to others.

For Arterial Ischemic Ulcers, Mixed Arterial and Venous Ulcers, and Diabetic Foot Ulcers, the most cited treatments in this study were *Calendula arvensis* (Marigold), *Citrus bergamia* (Bergamot orange), *Olea europaea* (Olive tree), *Plantago major* (White man’s footprint), *Salvia officinalis* (Sage), *Scrophularia canina* (Dog figwort), and *Vitis vinifera* (Common grapevine). Arterial and diabetic ulcers are prevalent among the elderly, and these plants are used to prevent infection and enhance skin regeneration.

*“My son brings me bergamot from the garden—we use the oil for my husband’s feet when they start to crack and go dark.”* (Marianna, female, 81 years old)

Bergamot’s antiseptic oil is particularly valued for preventing bacterial colonization in slow-healing wounds. Examining gender association, and given that arterial and diabetic ulcers disproportionately affect elderly men in rural settings, it is notable that the majority of herbal applications are cited by women. This likely reinforces the caregiver role of women in tending to their male family members.

### 5.3 Preparation methods and their symbolic meanings in healing

As for the methods of preparation of the herbs used, according to our key informants, is more than just a technical act—it is a culturally charged ritual, deeply embedded in the social fabric and the ethnobotanical knowledge passed down through generations. For the key informants included, the use of plant-based remedies is rooted in both practical necessity and symbolic significance, offering a rich insight into local healing practices that balance ancient wisdom with contemporary needs.

“*As my mother-in-law told me, using medicinal plants is not just about making medicine; it’s a tradition passed down through generations. It’s both practical and meaningful, helping people in ways that go beyond just healing.*” (Paolo, male, 65 years old)

The most common method of plant preparation in our sample is decoction, a process where herbs are boiled in water to extract their active compounds. This technique is the most frequently cited by the key informants, with plants such as *Calendula arvensis*, *Morus nigra*, and *Citrus bergamia* commonly used. The decoction process is seen not just to create a medicinal solution but as a symbolic act of extracting the plant’s “essence.” In the healing logic of the key informants, decoctions are typically employed to address conditions that require systemic treatment, such as circulatory disorders or chronic wounds. These ailments, which often demand long-term and comprehensive care, reflect the deeply ingrained belief that the essence of the plant can be best harnessed for the internal balance of the body (Pardo-De-Santayana et al., 2005). Another prominent method cited by the key informants is the preparation of infusions, where herbs like *Achillea millefolium* and *Petroselium crispum* are steeped in hot water, much like tea. Infusions are typically regarded as a gentler form of healing, favored for their nurturing properties. Often, elderly women prepare these infusions as part of long-term care, a form of preventive or slow-healing remedy rather than immediate treatment. In the cultural context of Calabria, this method is seen as both a therapeutic practice and a means of maintaining social bonds, as it is frequently prepared by family members for the care of loved ones. On the other hand, poultices, where plant material is directly applied to the skin or wounds, offer a more immediate and hands-on approach to healing. With plants such as *Plantago major* and *Achillea millefolium*, poultices are commonly used in cases of acute injury, often in folk first-aid scenarios. Interestingly, the use of poultices tends to be more prevalent among male users, suggesting a gendered pattern in herbal practice. Men, especially those engaged in physically demanding labor such as farming, may use poultices as a form of self-treatment for cuts, bruises, and other injuries. This pragmatic approach to healing aligns with the local understanding of folk medicine as a resource for immediate, practical relief (Van Andel et al., 2015). Finally, squashed pulp, where raw plant material is applied directly to the skin, represents a more unprocessed, “natural” approach to healing. Commonly made from *Citrus bergamia* and *Morus nigra*, this method preserves the integrity of the plant in its raw form. Symbolically, it reflects the region’s reverence for the natural world and its inherent healing powers. Squashed pulp is often used in urgent situations when there is insufficient time to prepare decoctions or infusions, highlighting its role as a quick and effective solution when immediate treatment is needed. Thus, the Calabrian key informants use of medicinal plants is not simply a technical process of healing, but a deeply symbolic practice intertwined with cultural beliefs, gender roles, and the practical demands of daily life. Whether through the slow, nurturing process of infusions, the systemic power of decoctions, or the immediate action of poultices and raw plant applications, these methods reflect a complex ethnomedical landscape where tradition, gender, and the exigencies of rural life converge.

Moreover, this is an important finding of this study, as symbolically, it reflects the region’s reverence for the natural world and its inherent healing powers. Squashed pulp is often used in urgent situations when there is insufficient time to prepare decoctions or infusions, highlighting its role as a quick and effective solution when immediate treatment is needed. Thus, the Calabrian key informants use of medicinal plants is not simply a technical process of healing, but a deeply symbolic practice intertwined with cultural beliefs, gender roles.

### 5.4 Cultural and historical significance: why do these practices persist?

Despite the growth and advancement of modern medicine, ethnomedical knowledge continues to thrive in the sample included in our study. This persistence is shaped by several key factors, deeply embedded in the social and cultural fabric of the region. These practices are not merely relics of the past but are actively maintained, transmitted, and adapted by local communities in response to both historical and contemporary challenges.

*When I was a child, I used to follow my grandmother into the hills. She would point to each plant and tell me what it was for—this one for stomach pain, that one for fever, another for wounds. It wasn’t just about the plants themselves but the way she spoke to them, how she gathered them with respect. These weren’t just weeds; they were part of our family’s medicine chest. We didn’t have doctors nearby, so we relied on what the land gave us. And now, I do the same with my granddaughter. I tell her the same stories, show her the same trails. It’s not something written in books—it lives in our memory, in our hands, in our way of life*. (Giulia, female, 89 years old)

A significant factor contributing to the enduring practice of ethnomedicine in Calabria is the limited access to formal healthcare in rural and remote villages. In these areas, access to modern healthcare services such as hospitals, medical professionals, and pharmaceutical drugs is often restricted due to geographical and infrastructural barriers. As noted by Etkin and Ross (2007), in many rural communities, healthcare systems remain insufficient, with long waiting times for consultations and a lack of access to essential treatments.

*“If someone has a fever in the night or a child gets injured while playing, we can’t just jump in the car and drive to a clinic—not when the roads are narrow, winding, and sometimes even blocked after heavy rain. We have to act quickly with what we have at home. My wife keeps dried herbs and ointments that her mother taught her to make. They’ve saved us more times than I can count. People think it’s old-fashioned, but when you’re this far from everything, you rely on tradition because it’s reliable. You can’t afford to wait for a prescription when someone’s in pain. You do what you’ve always done—you treat it yourself.”* (Tommaso, male, 76 years old)

The cost of pharmaceutical medications further exacerbates the problem, particularly for lower-income populations. In this context, herbal remedies, often passed down through generations, offer an accessible and economically feasible alternative, providing an immediate and culturally familiar solution.

*“I’ve never needed to go to a pharmacy for every little thing—why would I, when I can make a tisana with fennel or a compress with calendula from my own garden? These are plants I’ve known since childhood. My mother used them, and her mother before her. For stomach pain, I prepare an infusion of wild mint; for sore joints, I make a poultice with rosemary and olive oil. It’s not just about saving money—though for many of us, that matters—it’s about trust. These remedies have helped us all our lives. We know how to use them, when to gather them, how to combine them. It’s part of our rhythm, our seasons, our way of life. To us, it’s not ‘alternative’ medicine. It’s just medicine.”* (Giuseppina, female, 77 years old).

Ethnobotanical practices fill critical gaps, offering relief for conditions ranging from chronic ailments to acute injuries, where pharmaceutical treatments might be inaccessible or unaffordable (Tugume et al., 2016). Moreover, the persistence of traditional healing practices in Calabria is closely tied to the intergenerational transmission of ethnobotanical knowledge. This process not only ensures the survival of medicinal practices but also reinforces the cultural legitimacy of herbal medicine within the community. According to Pardo-De-Santayana et al. (2005), traditional knowledge is often maintained within family units and is passed down verbally or through observation, establishing a continuous link between generations. In Calabria, elderly women, who are often the primary carriers of herbal knowledge, play a crucial role in this transmission (Schulz et al., 2020).

*“When I was little, I would sit next to her while she sorted herbs on the kitchen table. She’d crush the leaves between her fingers and let me smell them—telling me, ‘This one soothes the stomach, that one helps you sleep, and this is for wounds.’ She didn’t just tell me the names—she told me the stories behind them, how her own mother had used them during the war when medicines were scarce. I learned by watching her, listening, helping. And now, when my daughter asks, I do the same. We go into the fields together, especially in spring, and I show her where the good wild chamomile grows, how to dry it properly, how to store it. It’s not just about healing; it’s about passing something on that holds our family together. These plants carry memory, and when I teach her, I feel like I’m keeping my grandmother alive too.”* (Rita, female, 79 years old)

The practices are deeply embedded in local identity, and this knowledge is not viewed as an “alternative” to modern medicine but as a living tradition, integral to community wellbeing. As Fantasma et al. (2024) highlights, the ability of local communities to preserve these traditions reflects the adaptive resilience of the community in maintaining a connection to their heritage while also responding to contemporary challenges.

The third contributing factor to the persistence of ethnomedicine in Calabria is the focus on sustainability and local resource utilization. The preference for local plants in medical treatments reflects a broader global movement toward ecological sustainability in healthcare. In many rural areas, communities rely on easily accessible and locally abundant plants, rather than costly imported pharmaceuticals.

*“Out here, we’re surrounded by plants our ancestors have used for generations. In spring and summer, I go out almost every day—up the slopes, near the olive groves, into the forest paths. I know where to find everything: St. John’s wort for burns, laurel for colds, juniper for the stomach. There’s no need to rely on expensive pills when we have these remedies growing all around us. And they’re not full of chemicals or made in a factory—we pick them, dry them, and use them with care. It’s not just about health—it’s about living in balance with nature. Using what’s local means we waste less, spend less, and stay connected to the land. For us, it’s a way of life, not a trend.”* (Pietro, male, 67)

This reliance on native flora reflects not only an ecological awareness but also an economically sustainable model of healthcare. Van Andel et al. (2015) discuss how the use of local plants minimizes the environmental footprint of medicine by reducing dependency on synthetic substances and foreign resources. This is particularly relevant in Calabria, where many medicinal plants grow abundantly in the wild and are integrated into the daily lives of the local population. By utilizing natural resources available within their environment, Calabrians practice a form of self-sufficiency that aligns with the principles of sustainability and environmental stewardship.

Considering similar findings in the existing literature, herbal medicine has long played a crucial role in wound healing across traditional medical systems, particularly in the Mediterranean, Latin America, and Africa. In the Mediterranean region, ethnobotanical surveys have documented nearly 1,000 plant taxa—predominantly from the Lamiaceae and Asteraceae families—used in wound management, highlighting a strong local knowledge system supported by historical and pharmacological evidence (Tsioutsiou et al., 2022). Phytochemical studies confirm that many of these plants possess antioxidant and regenerative properties that enhance fibroblast proliferation, angiogenesis, and tissue remodeling (Pathak and Mazumder, 2024; Raza et al., 2024). Similarly, in Latin America, a diverse pharmacopeia of indigenous and mestizo-origin plants—such as Ageratina pichinchensis, *Calendula officinalis*, and *Aloe vera*—have been validated in both preclinical and clinical studies for their wound-healing, anti-inflammatory, and antimicrobial effects (Salazar-Gómez and Alonso-Castro, 2022; Sánchez-Ramos et al., 2025). These practices are rooted in a holistic worldview that integrates spiritual, community, and ecological elements into healing processes (Baez et al., 2024). In African contexts, particularly in regions such as Mali and Nigeria, wound care remains a cornerstone of traditional medicine, with plants like *Aloe macrocarpa* and Terminalia avicennioides employed for their antiseptic and antioxidant capabilities (Inngjerdingen et al., 2004; Builders and Builders, 2016; Gulumian et al., 2018). Across these regions, traditional herbal medicine not only complements conventional therapies but also offers culturally embedded, accessible, and biologically active solutions for wound care that merit continued scientific exploration.

Considering the analysis of plant efficacy, in several cases, there is a notable convergence between local perceptions and the pharmacological properties recognized in contemporary biomedical literature. For example, *Achillea millefolium* (yarrow), widely used for its ability to “draw out bad blood” and reduce swelling, has documented anti-inflammatory and antimicrobial effects that support its use in wound healing (Benedek et al., 2007). Similarly, *Calendula arvensis* (marigold) and *Plantago major* have been shown in clinical and preclinical studies to enhance fibroblast proliferation and tissue regeneration (Zhakipbekov et al., 2023; Al-Adwan et al., 2025). However, for some other plants cited by informants—such as Loranthus europaeus or *Marrubium vulgare*—the biomedical literature remains limited or inconclusive, despite their longstanding use in folk medicine, as remain still underexplored in wound research. This gap illustrates how local knowledge can guide the identification of promising species for future pharmacological research. Furthermore, in a few cases, the cultural rationale for plant use—such as “removing heat” or “bringing out bad blood”—may not align with biomedical etiologies but reflects a coherent symbolic logic within local ethnomedical systems. These cases illustrate the need for integrative frameworks that respect both empirical efficacy and culturally embedded meanings in wound care.

Moreover, considering broader sociocultural dynamics, while our participants did not explicitly reference broader structural factors such as migration, economic decline, or shifts in family structure, we recognize that these dynamics are highly relevant to the sustainability of ethnomedical knowledge systems in Calabria. The region’s ongoing demographic challenges—marked by youth outmigration, aging populations, and economic marginalization—likely influence both the transmission and daily practice of herbal medicine in subtle yet significant ways. For instance, the erosion of extended family living arrangements and the depopulation of rural areas may weaken intergenerational pathways through which matrilineal healing knowledge has traditionally been passed down. Similarly, economic precarity may both reinforce reliance on low-cost traditional remedies and simultaneously limit opportunities for younger generations to engage with and preserve these practices. Although these themes did not emerge directly in our dataset, they remain critical to understanding the long-term resilience of vernacular healing systems.

## 6 Conclusion

This ethnographic study sheds light on the enduring vitality of herbal medicine in the management of chronic wounds among patients in Calabria, southern Italy. Far from being an outdated or marginal practice, the use of medicinal plants emerges here as a living tradition, deeply rooted in familial memory, shaped by gendered knowledge transmission, and adapted to meet the evolving healthcare needs of aging, rural populations. One of the most salient findings of this research is the pivotal role of women as custodians of herbal healing knowledge. In the Calabrian context, the use and transmission of medicinal plant practices are predominantly matrilineal: grandmothers, mothers, and daughters form a chain of intergenerational knowledge that binds ethnomedical wisdom to caregiving roles within the family. This gendered structure not only reinforces women’s agency in domestic healthcare but also positions them as cultural stewards who mediate between tradition and necessity. The dataset, which shows significantly more female citations of herbal use compared to males, reinforces this dynamic. While men do participate — particularly in the application of poultices for work-related injuries or self-treatment — it is women who sustain the broader ethnobotanical framework for chronic wound care, particularly for complex and long-term conditions like venous ulcers or diabetic foot complications. In parallel, the findings reveal that herbal medicine persists not in isolation from, but in response to, structural deficiencies in the biomedical system. Many participants turn to plant-based therapies not out of ideological opposition to conventional medicine, but due to pragmatic barriers: lack of access to specialists, long waiting times, the high cost of treatments, and the limited presence of vascular clinics in rural Calabria. Herbal medicine thus fulfills a dual role: it is both a therapeutic alternative and a culturally resonant form of health self-management. This is particularly evident in the treatment of chronic ulcers — conditions known for their slow healing trajectory, recurrence, and resistance to standard protocols. In this landscape of therapeutic frustration, herbal treatments are perceived as empowering, offering patients a degree of control, familiarity, and low-cost accessibility. Another crucial finding is the selective and context-specific use of plants for different types of chronic wounds. The participants demonstrated not only deep familiarity with local flora but also a form of cultural logic in plant selection, tied to the perceived etiologies of wounds. For venous leg ulcers and related conditions like lipodermatosclerosis and venous eczema, plants such as *Achillea millefolium*, *Cichorium intybus*, *Marrubium vulgare*, and *Equisetum arvense* were frequently cited — species known for their anti-inflammatory, antimicrobial, and circulatory-enhancing properties. This suggests that local knowledge aligns, at least partially, with pharmacological profiles recognized in scientific literature. In contrast, for arterial ulcers and diabetic foot ulcers — conditions associated with ischemia and systemic metabolic dysfunction — informants frequently cited the use of *Calendula arvensis*, *Citrus bergamia*, *Plantago major*, and *Vitis vinifera*. These species were valued for their regenerative and antiseptic properties, as well as for their ability to stimulate tissue repair in wounds with poor perfusion. Remarkably, while diabetic and ischemic ulcers are more prevalent among elderly men in the study, it was predominantly women who reported the preparation and administration of the remedies — a finding that again underscores their caregiving centrality in chronic illness contexts. The methods of preparation reveal further insights into the symbolic and practical dimensions of healing. Decoctions and infusions are not merely biochemical processes for extracting active compounds — they are ritualized acts that connect the healer with the plant and the patient. Decoctions, involving prolonged boiling, are associated with the extraction of a plant’s essence and are perceived as stronger, systemic treatments, often used for entrenched or internal conditions. Infusions, by contrast, are gentler and associated with nurturing, ongoing care — often made by older women for their spouses or children. Poultices and squashed pulps, on the other hand, suggest a more immediate, hands-on mode of healing. These methods are often employed in acute or visible wounds, especially by male informants. This division not only indicates gendered modes of practice but also points to a vernacular taxonomy of treatment intensity, urgency, and appropriateness. The use of raw plant materials in squashed pulp or direct application is especially revealing of a Calabrian ethos of proximity to nature — the belief that healing can emerge from minimal intervention and maximum intimacy with the natural world. Finally, the resilience and cultural persistence of these practices should not be underestimated. In an age of medical globalization and pharmaceutical dominance, the Calabrian model illustrates how traditional medicine continues to coexist with modern healthcare. The persistence is driven by both structural necessity (healthcare gaps, economic constraints) and cultural identity. For many of the informants, especially the elderly, herbal medicine is not merely a therapeutic choice but a form of continuity — a connection to the ancestors, to the land, and to ways of life that resist erasure. The symbolic dimensions of healing, the stories associated with each plant, and the ritualized modes of preparation form an intangible heritage that is as crucial as any pharmacological effect. This study contributes to the understanding of how ethnomedical knowledge remains deeply embedded in local ecologies, gender dynamics, and healthcare behaviors. Herbal medicine in Calabria is more than a survival strategy; it is a social institution — one that fills therapeutic gaps, affirms cultural belonging, and reconfigures care in the absence or inadequacy of biomedical solutions. For policymakers and health professionals, these findings underscore the importance of engaging with local knowledge systems not as competitors to modern medicine but as potential allies. The future of chronic wound care, particularly in underserved areas, may lie not in the displacement of traditional practices but in their integration — ethically, respectfully, and with a deep understanding of their cultural roots.

Building on the ethnographic insights of this study, we propose that meaningful integration of traditional knowledge and biomedical practice in Calabria could take the form of community-based initiatives that include local knowledge holders—particularly experienced elder women and local herbalists—in patient education and self-care support programs. For example, community health worker (CHW) models could be adapted to rural Calabrian contexts by training culturally embedded mediators who are familiar with both local ethnobotanical practices and basic biomedical wound care. These mediators could serve as trusted liaisons between patients and healthcare providers, helping to translate medical advice into culturally resonant practices while also ensuring that traditional remedies are used safely and complementarily. Such initiatives would not only improve patient adherence and therapeutic outcomes but also affirm the cultural legitimacy of local healing traditions. Importantly, any integration must be grounded in mutual respect, participatory dialogue, and ethical safeguards to avoid the extraction or devaluation of community-based knowledge systems. Future policy and health interventions should prioritize these pluralistic and collaborative approaches as part of a broader strategy to address healthcare disparities in underserved regions like Calabria.

## Data Availability

The original contributions presented in the study are included in the article/supplementary material, further inquiries can be directed to the corresponding authors.
